# Neck-shaft angle measurement in children: accuracy of the conventional radiography-based (2D) methods compared to 3D reconstructions

**DOI:** 10.1038/s41598-022-20832-1

**Published:** 2022-10-03

**Authors:** Ádám Tibor Schlégl, Viktória Nyakas, Dániel Kovács, Péter Maróti, Gergő Józsa, Péter Than

**Affiliations:** 1 Department of Orthopaedics, Medical School, University of Pécs, Akác str. 1., Pecs, 7632 Hungary; 2 3D Printing and Visualization Centre, University of Pécs, Medical School, Boszorkány str. 2., Pecs, 7624 Hungary; 3 Division of Surgery, Traumatology and Otorhinolaryngology, Department of Pediatrics, Medical School, University of Pécs, József A str. 7, Pecs, 7623 Hungary

**Keywords:** Signs and symptoms, Anatomy, Musculoskeletal system

## Abstract

Aim of this study was to examine the accuracy of widely used conventional radiography-based (2D) neck-shaft angle measurements compared to 3D reconstruction. In our retrospective study, EOS 2D/3D images of 156 patients (312 limbs) were selected from our database (4–16 years old: 6 girls and 6 boys/year), where no pathology was revealed. Using the 2D modality of the EOS method neck-shaft angle was measured using the “biggest diameter” and “circle fitting” techniques to define the femoral neck axis and 1/3, 1/2 and full femur to determine the femoral shaft axis. EOS 3D reconstructions of same images were also performed and a comparison of 2D and 3D results was made. We did not find any significant difference between accuracy of the four examined 2D methods, although the deviation between 2 and 3D results was considerable (average difference: 5.11–5.58°, *p* < 0,001). In 31% of the cases, difference was more than 10°. Only femoral torsion showed significant influence on the difference (correlation coefficient: 0.380, *p* < 0.001). We did not find a clinically significant difference between the examined 2D methods, although their accuracy was highly questionable compared to 3D results. We suggest using any 3D imaging method for surgical planning and in uncertain cases.

## Introduction

The neck-shaft angle (NSA), which is also known as the collodiaphyseal angle or caput-collum-diaphyseal angle is one of the most important parameters to describe the proximal femur, due to its impact on paediatric and adult hip pathologies (such as coxa vara and valga, hip dysplasia, femoral impingement, osteoarthritis, risk of femoral neck fracture, etc.) and surgical planning. Still, there is no agreement on the measurement’s methodology^1^.

The NSA can be described as the angle between the femoral neck and femoral shaft axis in the plane of the femoral neck, although the exact definition and determination of the femoral neck and femoral shaft axis are still an open question. The femoral neck axis (FNA) can be defined as the line between the center of the femoral head and the femoral neck; however, their exact definition varies in the literature. The femoral shaft axis (FSA) can also differ pending on the part of the femur considered^1,2^. Boese et al.^3^ suggested to use a modified FNA, the line between the center of femoral head and the horizontal tangent of the FSA in the level of the lesser trochanter’s apex.

**Figure 1:**
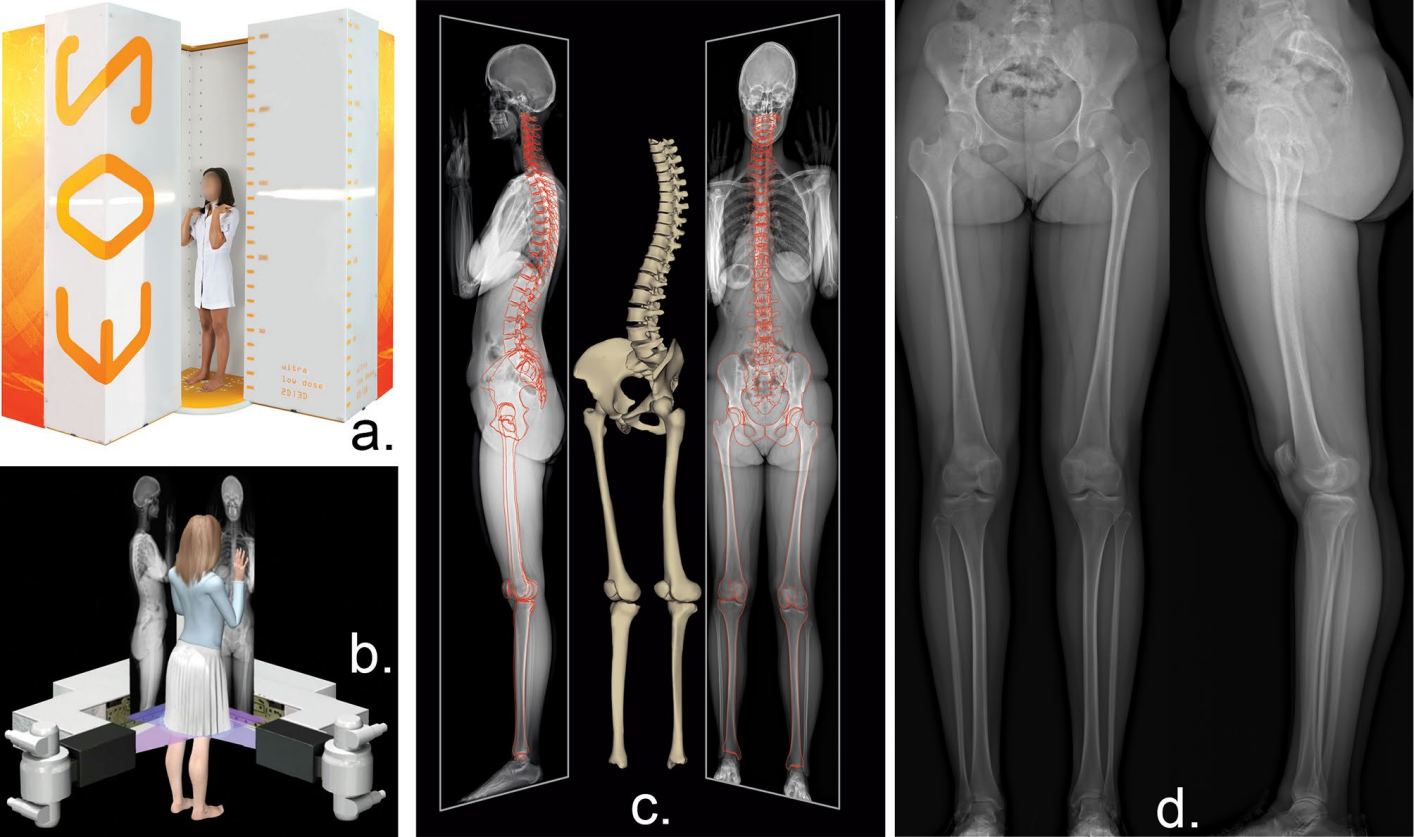
The EOS 2D/3D Imaging System. (**a**) The EOS 2D/3D Imaging System and the typical positioning during examination (source: www.eos-imaging.com). (**b**) The basic operation of the imaging system: upright biplanar slot-scanning X-ray imaging (source: www.eos-imaging.com). (**c**) 3D reconstruction opportunities of the lower limb and spine (source: www.eos-imaging.com). (**d**) Example of a typical EOS 2D/3D lower limb image-pair used in this study.

The most often used radiological modality to measure NSA is the anteroposterior plain radiograph in a neutral or internally rotated position^1^. There are a few methods using two plain radiographs and mathematical formula, but the additional radiation and long application time prevent their wide use^4,5^. CT and MRI are suitable to assess true NSA, since the measurement can be done in the plane of the neck. However, many protocols exist, and the determination of the anatomical landmarks can be challenging^6–8^. The EOS system can deliver low-dose stereoradiographic images in a weight-bearing position with the opportunity of surface 3D modelling. The system proved to be reliable to measure lower limb anatomical and biomechanical parameters^9–12^, even in children^13–15^ (Fig. 1).

Although the effect of femoral torsion and rotation/flexion malpositioning on the measured NSA is widely known, most of the studies are based on ex vivo mathematical analysis or modelling of fully developed hips.Wordie et al. examined the effect of rotation and flexion on the head-shaft angle using a dry bone model and found acceptable accuracy (< 5°) between 20° internal and 40° external rotation, as well as under 60° of flexion^16^. Kay et al. used mathematical modelling and cadaver studies to define the safe zone to measure NSA. The bias of measurement was found to be less than 5° between 20° external and 50° internal rotation. They found 10° internal rotation as the most reliable position for measurement^17^. In contrast Boese et al.^1^ did not find a difference between the non-corrected and rotation-corrected results in their review. O’Connor et al.^18^ found that lower external rotation (< 30°) with flexion decreased the NSA, but in the case of higher external rotation (> 30°), flexion increased it. Bhashyam et al. examined the influence of the limb’s position on the fixation of femoral neck fractures using mathematical models and sawbones. They found that, if the rotational error was more than 10°, even 5° of flexion or extension can cause more than 10° bias in the NSA^19^.

In the reviewed international literature, only Bizdikian et al. examined the accuracy of the 2D-based NSA measurement methods and the effect of the positional error in a younger population. In their 3D CT reconstruction-based study, they enrolled 9 adults and 8 adolescents (9–15 years old) and found the circle-fitting method as the most accurate^2^.

Despite its clinical significance and widespread use in paediatric orthopaedics, we have not found any paper examining the accuracy of the 2D measurement methods in children, especially in the clinical setting. The aim of this study to test the accuracy of the widely used NSA measurement method on plain radiographs in children.

- Question 1: Which is the most accurate from the widely used plain radiograph-based NSA measuring methods in children compared to 3D measurement?
- Question 2: What is the difference between the true (3D) and plain radiograph-based (2D) NSA? Is it clinically significant?
- Question 3: We have examined 19 anatomical and biomechanical parameters as potential influencing factors or biases of this difference.

## Materials and methods

Study design: Retrospective diagnostic study.

### Examined population

Sample size calculation suggested a sample group of 136 participants to prove 2° of difference (average NSA was set to 129.88 ± 5.09 based on our previous studies^13,14,20,21^, alpha: 0.05, beta: 0.1).

We reviewed the EOS picture pairs from our database that were collected during routine clinic work between 2007 and 2021. We selected the radiographs representing the population 4–16 years old, where no biomechanical pathology of the lower limb was found, and there were no lower limb surgeries or developments influencing disease in the history of the patient. We randomly chose six girls and six boys from each year, resulting in 156 image pairs (312 limbs). All investigations were performed with an orthopaedic indication (joint pain of unknown origin) in an upright weight-bearing position (non-step-forward) (Fig. 1).

### Neck-shaft angle measurement

The NSA is defined as the angle between the axis of the femoral neck and the femoral shaft.

The AP view of the EOS image pairs and SterEOS software toolbox (v.1.8.5.57R, EOS Imaging, Paris, France) were used to evaluate the NSA.

**Figure 2:**
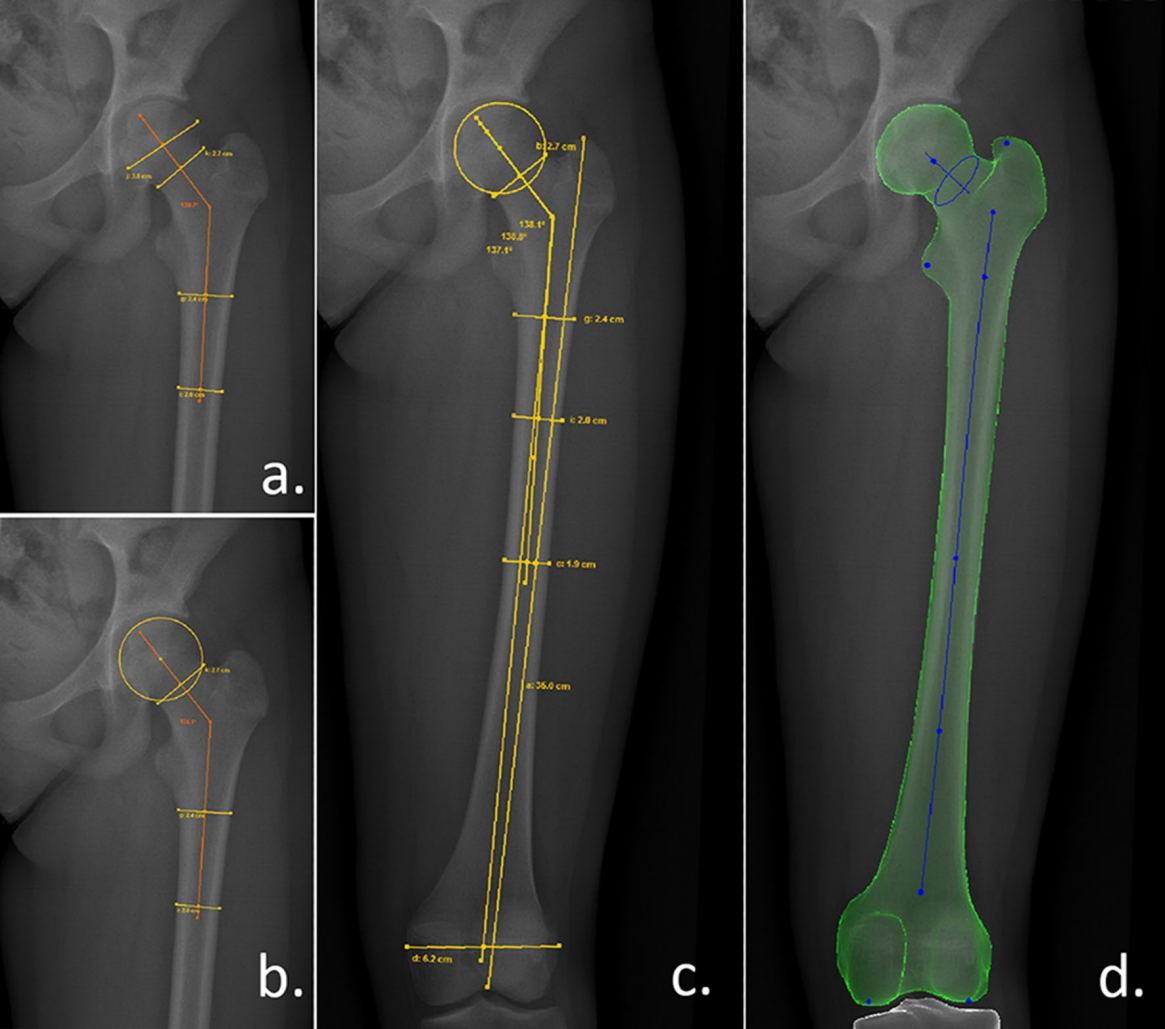
Neck-shaft angle measurement methods (OM, 11 years old, girl). (**a**) Biggest diameter-1/3 femur: 139.7°. (**b**) Circle fitting-1/3 femur: 138.1°. (**c**) Circle fitting-1/2 femur: 138.8°, Circle fitting-full femur: 137.1°. (**d**) SterEOS 3D reconstruction of the femur-true NSA: 123.6°.

We have defined the FNA using the following methods (Fig. 2):

- Circle fitting method: The centre of the circle fitted to the femoral head’s contour and the midpoint of the femoral neck’s smallest diameter (Fig. 2a).
- Biggest diameter method: The midpoint of the femoral head’s biggest and the femoral neck’s smallest diameter (Fig. 2b).
- Modified NSA, as described by Boese et al.: The centre of the circle fitted to the femoral head’s contour and the femur-shaft axis in the height of the lesser trochanter’s apex^3^.

We have assigned the FSA by the midpoint of the femur’s smallest diameter 1 cm under the lesser trochanter and at 1/3, 1/2 or distal end of the femur (Fig. 2c).

We performed the 2D measurements manually. The circle fitting was made using three points fitting method: three markers were passed by hand to the contour of the femoral head defining a circle, the software gave the centre of circle automatically (Fig. 2b). The observers applied the ruler tool of the software to find the biggest distance in the femoral head (the software gave the halfway point of the segment automatically) (Fig. 2a). To define the midpoint of the femoral neck and shaft, also the ruler tool was used: the observers manually sought for the smallest distance in the defined level between the two sides of the bone’s contour (Fig. 2a–c).

### 3D reconstruction

SterEOS 3D (v.1.8.5.57R, EOS Imaging, Paris, France) reconstructions were also performed using the same picture pairs. In this process, a surface model of the femur is generated using 16 different landmarks defined by a technician. Based on the model, the software automatically calculates the NSA and 19 other parameters (the detailed description of the reconstruction process, the list and definitions of the parameters are in Supplementary material 1) (Figs. 1c, 2d).

### Study protocol

First intra- and interobserver reliability analyses were performed on each 2D-based measurement method to test the observers’ ability to use the techniques and the measurement protocol’s reproducibility. A total of 10 pictures were randomly selected and analysed by three observers (two junior orthopaedic specialists and a Ph.D. candidate), on three different days with a minimum of 1 week in between, three times. The results were evaluated using Winer’s criteria^22^. After the reliability analysis, we excluded the modified NSA measurement as reported by Boese et al. due to difficulties defining the apex of the lesser trochanter in younger ages.

After that, we measured the NSA using the AP views of the EOS images. The NSA was defined using the biggest diameter-1/3 femur, circle fitting-1/3 femur, circle fitting-1/2 femur and circle fitting-full femur.

Parallel with that, SterEOS 3D modelling using the same pictures were performed by a senior orthopaedic surgeon with 11 years’ experience in lower limb reconstruction. The two working groups worked separately and did not know each other’s results. The diagram of the study protocol is presented in Supplementary material 2.

### Statistical analysis

All statistical evaluations were performed with IBM SPSS™ (v 27. IBM Corp., Armonk, NY, USA). For randomization, the RAND.BETWEEN formula of Microsoft Excel software (v 2105. Microsoft Corp., Redmond, WA, USA) was used. The Shapiro–Wilk test was used to examine normality, paired-sampled and independent-samples t-test to compare groups, Pearson and Spearman correlations to examine the possible influencing factors and Cronbach’s alpha for reliability studies. A *p* value < 0.05 was considered statistically significant. The data in the text are given in average ± S.D.

### Ethical approval and informed consent

The study was conducted according to the guidelines of the Declaration of Helsinki, and approved by the Institutional and Regional Ethical Review Board of University (7607-PTE2019, date of issue: 01.02.2019.). At the time of initial radiological evaluation, written consent was collected for future retrospective studies. Informed written consent at the time of imaging was attained from all individuals, or their guardians.

## Results

The 2D measurements and 3D reconstructions were successful in all cases. All examined parameters showed normal distributions, except for pelvis axial rotation.

All 2D measurement methods performed excellent (Cronbach’s alpha > 0.9) on intra- and interobserver studies (Supplementary material 3).

### Accuracy

Based on the results of 3D reconstructions, the NSA decreased from 132.48° (4-year-olds) to 127.61° (16-year-olds). In contrast, all 2D measurement methods gave us significantly higher results: 138.49–140.25° at the age of 4 years and 129.00–130.42° at the age of 16 years (*p* < 0.001 in all cases) (Table 1).

**Table 1:** Average NSA and femoral torsion results per 3D reconstruction and each 2D NSA measurement method (mean ± S.D., degrees).

Age (year) n = 24 limbs/year	3D measurement	Biggest diameter—1/3 femur	Circle fitting—1/3 femur	Circle fitting—1/2 femur	Circle fitting—full femur	Femoral torsion
4	132.48 ± 3.60	139.80 ± 4.11	138.49 ± 3.88	140.25 ± 5.35	140.10 ± 5.38	24.23 ± 9.76
5	131.28 ± 5.11	138.83 ± 7.49	137.49 ± 6.87	138.88 ± 7.78	138.65 ± 7.59	24.57 ± 12.34
6	130.28 ± 4.60	138.08 ± 7.62	138.81 ± 7.49	138.63 ± 6.78	138.92 ± 6.47	24.85 ± 13.49
7	130.20 ± 7.28	135.23 ± 7.41	136.00 ± 7.34	136.44 ± 7.95	138.82 ± 8.51	24.56 ± 9.25
8	130.53 ± 6.28	135.42 ± 6.88	135.75 ± 7.27	134.72 ± 7.25	135.38 ± 6.94	22.02 ± 9.59
9	130.48 ± 5.88	135.01 ± 6.03	134.16 ± 6.97	133.31 ± 6.48	133.53 ± 6.59	22.71 ± 6.67
10	130.89 ± 5.26	135.53 ± 5.65	136.06 ± 6.18	135.05 ± 5.12	135.27 ± 6.43	21.73 ± 10.08
11	129.25 ± 4.76	134.13 ± 7.02	133.85 ± 6.85	133.19 ± 5.36	133.25 ± 5.29	20.51 ± 12.75
12	129.34 ± 4.13	133.30 ± 5.03	133.19 ± 5.78	132.20 ± 5.04	132.82 ± 4.54	21.28 ± 10.23
13	128.97 ± 4.27	133.83 ± 3.37	133.29 ± 3.40	132.65 ± 3.63	133.49 ± 3.85	21.63 ± 6.19
14	128.97 ± 4.62	131.28 ± 7.34	130.53 ± 7.62	129.73 ± 7.79	130.15 ± 7.77	18.18 ± 11.48
15	128.11 ± 4.44	133.39 ± 6.27	131.87 ± 5.93	131.03 ± 5.21	131.71 ± 5.17	19.80 ± 10.20
16	127.61 ± 3.73	130.42 ± 6.22	129.19 ± 5.45	129.00 ± 5.95	129.84 ± 5.95	16.60 ± 11.72
SUM (n = 312 limbs)	129.88 ± 5.09	134.95 ± 6.74	134.51 ± 6.86	134.24 ± 7.02	134.51 ± 6.96	21.78 ± 10.56

**Figure 3:**
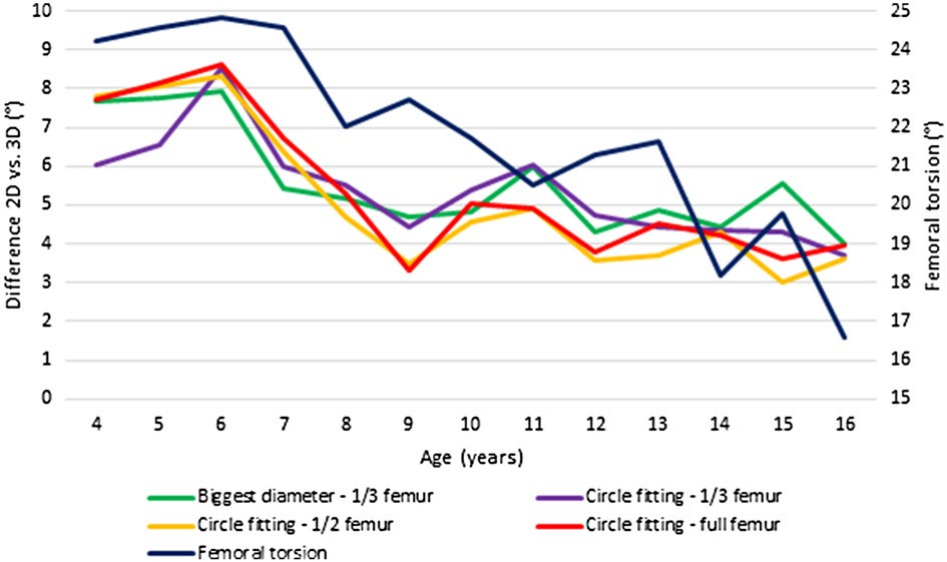
The average difference of the 3D reconstruction and the plain radiography-based NSA measurement results, in addition the average femoral torsion.

**Figure 4:**
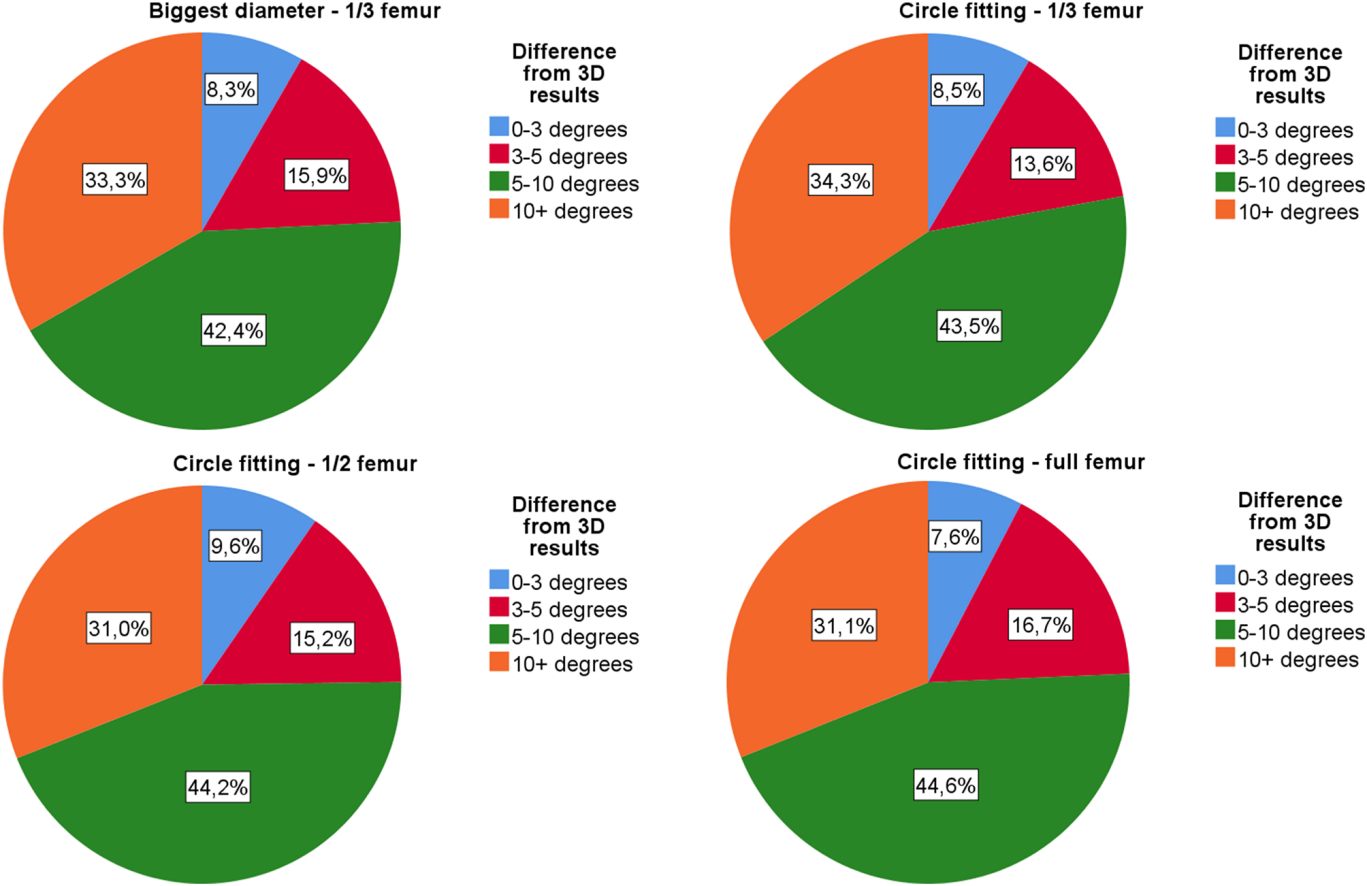
Pile chart of the difference between the 3D reconstructions and each 2D measurement result. Blue—the absolute difference between the 2D and 3D results is less than 3° (7.6–9.6%). Red—the absolute difference between the 2D and 3D results is between 3–5° (13.6–16.7%). Green—the absolute difference between the 2D and 3D results is between 5–10° (42.4–44.6%). Yellow—the absolute difference between the 2D and 3D results is more than 10° (31.0–34.3%).

We found more than 5° of average difference between the 3D and 2D data in each method. The most accurate technique was the circle fitting-1/2 femur method with 5.11 ± 3.99° average difference (3.02–8.34°). In 24.7% of the cases (77/312), the difference was less than 5°, and in 31.0% of the cases (97/312), the difference was more than 10°. The most inaccurate was the biggest diameter-1/3 femur method, with an average 5.58 ± 4.12° difference (3.98–7.91°). In 24.4% of the cases (76/312), the difference less than 5°, and in 33.3% of cases (104/312), the difference was more than 10° (Figs. 3, 4, Supplementary material 4 and 5).

We did not find any gender specific difference regarding the 3D NSA results, femoral torsion values and difference between the 3D and 2D data (Supplementary material 4).

With regard to reliability analysis, we obtained good results in each test compared to the 3D reconstruction (Cronbach’s alpha: biggest diameter-1/3 femur: 0.814; circle fitting-1/3 femur: 0.822; circle fitting-1/2 femur: 0.825; circle fitting-full femur: 0.826).

### Influencing factors or biases

To examinate the influencing factors, we used the circle fitting-1/2 femur method, since we found that as the most accurate 2D method in the previous step of the study. Generally, with the advancement of age, the accuracy of the measurements improved. The difference between the 2D and 3D results decreased from 6.05 to 7.80° at the age of 4 years to 3.60–3.98° at the age of 16 years (Pearson correlation coefficient: − 0.446, *p* < 0.001).

From the 19 examined anatomical and biomechanical parameters, only femoral torsion showed significant correlation with the difference (Pearson correlation coefficient: 0.380, *p* < 0.001) (Fig. 2).

Pelvis axial rotation can predict positional errors, since it shows the angle between the pelvis and plane of the detector. In our sample, the average rotation was − 0.18 ± 3.31° (range: − 11.81 to 12.70°). In 86.5% of the cases (270/312), the deviation was less than 5°. There was no significant difference between the accuracy of the groups with more and less than 5° of rotation. Based on nonparametric correlation tests, there was no significant relationship between the difference of the 2D and 3D results and pelvis axial rotation (*p* = 0.053).

## Discussion

It is a well-known fact that the complex geometry of the proximal femur, the possible positional errors, and the nature of the plain radiographs (enlargement, elongation/foreshortening, superimposition etc.) affect the accuracy of the NSA measurement^23^. Despite its clinical significance and widespread use in paediatric orthopaedics, we have not found any paper examining the accuracy of the 2D measurement methods in children, especially in routine daily practice. The cause of this insufficiency could be the former lack of the proper radiological method to examine the hip in 2D and 3D at the same time with acceptable radiation.

### Question 1: Which is the most accurate from the widely used plain radiograph-based (2D) NSA measurement methods in children compared to 3D measurement?

We did not find a significant difference between the accuracy of the four examined methods. The circle fitting method proved to be slightly more precise than the biggest diameter method (5.35° vs. 5.58° average difference). Comparing the different definitions of FSA, taking account 1/2 of the femur was found to be the most accurate (average difference of 5.11°). Although it did not significantly deteriorate the correctness, we defined the FSA based on only 1/3 of the femur, which is usually represented on standard hip radiographs.

Bizdikian et al. also found the circle fitting technique as the most accurate, whereas their methodology can hardly compare to this study: they used a post-hoc 2D reconstructions of 3D CT images made in lying (non-weight bearing) position for the conventional (2D) measurements; they rotated the reconstructions manually, what can not simulate the positional errors because of the patient’s movement or bad posture; they used axially corrected CT 3D reconstruction to measure true (3D) NSA values; their study population was significantly smaller (17 patients)^2^.

Similarly to previous studies, we found excellent intra- and interobserver reliability in all methods^1,2,24,25^.

### Question 2: What is the difference between the true (3D) and plain radiograph-based (2D) NSA? Is it clinically significant?

We found a clinically relevant difference between the 3D and 2D results. The average bias was over 5° in all methods. In the younger ages (4–6 years old), the average deviation varied between 6.05° and 8.63°, which declined to 3.02–5.58° in the older group (14–16 years old). Even with the most accurate technique, only 24.8% of the cases had less than 5° difference, and 31% of cases had more than a 10° difference. Our results of the circle fitting-1/3 femur method in the population 14–16 years old harmonized with the data published by Bizdikian et al.^2^.

Chung et al. examined 36 patients with cerebral palsy in their study, in which—among others—they evaluated the validity of conventional NSA measuring method (circle fitting) compared to CT 3D reconstructions-based evaluation. They found an average 4.0 ± 3.4° difference between the 2D and 3D results, with 90% of the measurements being within 10°. This smaller difference can be explained by the specific, pathological study population (higher femoral torsion and NSA are typical in this condition), the relatively small number of the patients, the way of the conventional imaging’s positioning (they tried to compensate the high femoral torsion with internal rotation) or the difference between the involved imaging modalities^25^.

More dry bone, sawbone, cadaver or modelling study investigated to potential effect of malposition on NSA measuring. Wordie et al. and Kay et al. found quite big safe zone to measure NSA with acceptable accuracy (< 5°)^16,17^. In contrast, Bhashyam et al.^19^ stated that 10° rotational error with only minimal flexion or extension can cause more than 10° difference. It is difficult to compare their results to this study since they have used adult size bones or models, simple one- or two-dimensional positional differences and non-clinical setting.

### Question 3: Potential influencing factors or biases

We found femoral torsion was the only parameter showing significant correlation with the difference between the 2D and 3D results. Based on the pelvis axial rotation results, there was no significant bias because of patient positioning error.

## Limitations

All observers are highly trained and experienced in the field of radiological measurements and have worked together for years, which could potentially positively influence the reliability data. Unlike conventional radiographs, the EOS imaging system does not suffer from horizontal distortion and the vertical distortion is also computer-corrected. Another limitation is this is a single-centre study examining only a Middle-European Caucasian population. Although we have not found any pathology, the patients had an orthopaedic complain at the time of examination. By including only a population without orthopaedic disorders, a pathological hip morphology could potentially influence the results. We could measure the angle between the patient, source and detector but were not able to exclude the bias from the inaccurate positioning caused by femur rotation. The methodology of this study is linked to the EOS 2D/3D imaging system, therefor it can be reproduced only in those institutions, where this technique is available.

## Conclusion

In conclusion, the accuracy of the NSA measurement on standard AP hip radiographs in children is highly questionable. The accepted normal range of the NSA is about 10°, and we found more than 10° of difference between the measured and true angle in 31% of the cases. Thereby we need to conclude, we cannot trust in conventional radiographs to measure neck-shaft angle in children.

Although the advantages of the conventional radiography must be considered: widely accessible, cheap, fast imaging, weight bearing position can applied (instead of CT or MRI examination), no need of sedation (instead of MRI or CT examination) and fast NSA measuring. EOS imaging can provide an acceptable 3D measurement alternative, but it is available only in about 400 facilities worldwide (per EOS imaging Ltd.) and the 3D reconstruction is time consuming. The relatively high radiation dose of CT imaging, the limited accessibility and long examination time of MRI confine their use in everyday practice. Because of these limitations of the 3D measurement methods the regular use of conventional radiography must be accepted to measure NSA, but its uncertainty needs to be taken into account. We suggest using any 3D technique (EOS, CT or MRI) in any uncertain case and for surgical planning.

The circle fitting-1/2 femur method proved to be the most accurate, even though there was no clinically significant difference in the four examined conventional radiography-based (2D) methods.

## Supplementary Information

Supplementary Information 1.

Supplementary Information 2.

Supplementary Information 3.

Supplementary Information 4.

Supplementary Information 5.

## Data Availability

The datasets generated and/or analysed during the current study are not publicly available due data protection of the participants but are available from the corresponding author on reasonable request.
