# Author Correction: Neck-shaft angle measurement in children: accuracy of the conventional radiography-based (2D) methods compared to 3D reconstructions

**DOI:** 10.1038/s41598-022-26238-3

**Published:** 2022-12-27

**Authors:** Ádám Tibor Schlégl, Viktória Nyakas, Dániel Kovács, Péter Maróti, Gergő Józsa, Péter Than

**Affiliations:** 1 Department of Orthopaedics, Medical School, University of Pécs, Akác Str. 1, Pecs, 7632 Hungary; 2 3D Printing and Visualization Centre, Medical School, University of Pécs, Boszorkány Str. 2, Pecs, 7624 Hungary; 3 Division of Surgery, Traumatology and Otorhinolaryngology, Department of Pediatrics, Medical School, University of Pécs, József A Str. 7, Pecs, 7623 Hungary

Correction to: *Scientific Reports* 10.1038/s41598-022-20832-1, published online 03 October 2022

The original version of this Article contained an error in Affiliation 2, which was incorrectly given as ‘3D Printing and Visualization Centre, University of Pécs, Boszorkány str. 2., Pecs, 7624, Hungary’. The correct affiliation is listed below:

3D Printing and Visualization Centre, University of Pécs, Medical School, Boszorkány str. 2., Pecs, 7624, Hungary.

The original Article has been corrected.

