# Supplementary material for: Neck-shaft angle measurement in children: accuracy of the conventional radiography-based (2D) methods compared to 3D reconstructions
Source: Sci Rep. 2022 Oct 3;12:16494. doi: 10.1038/s41598-022-20832-1 (PMC9529964; doi:10.1038/s41598-022-20832-1)
Supplement: Supplementary file 1 — Supplementary Information 1. [file 41598_2022_20832_MOESM1_ESM.pdf]

Supplementary material 1.

EOS Lower limb Full 3D reconstructions

The EOS 3D reconstruction of the lower limb can be performed using a matching EOS image-pair in the SterEOS 3D software (v.1.8.5.57R, EOS Imaging, Paris, France) (Figure 1/c).

The reconstruction is composed of three steps:

In the first step, the pelvic parameters must be defined. The observer appoints manually the plateau of the sacrum, the acetabuli and sacroiliac joints.

In the second step, the observer needs to define 16 landmarks of the femur and tibia by hand (femoral head contour, midpoint of the femoral diaphysis, femoral neck's edges, centre of the proper ankle joint etc.). All landmarks need to fitted to both images (anteroposterior and lateral) parallelly. The newest version of the software places some of the points automatically, the observer only needs to confirm them. Based on these markers the software generates a surface model of the femur, tibia and fibula.

In the third step, the observer oversees and – if required – adjusts the fitted universal model to the image-pairs. The software provides several correction opportunities including moving, resizing and shape-changing of the relevant anatomical regions (femoral head and neck, trochanters, femoral condyles, ankle joint etc.).

At the end of reconstruction, the observer needs to approve the fitted model, whereupon the software calculates 20 parameters of the pelvis, femur and tibia automatically (Figure 2/d).

Calculated parameters

The list and definitions of the parameters automatically calculated by the SterEOS 3D software include:

- Length of the femoral mechanical axis (femur length) (mm);
- Length of the tibial mechanical axis (tibia length) (mm);
- The length of the lower limb (limb length) (mm);
- Femoral head diameter (mm);
- Neck length (the distance between the centre of the femoral head and the proximal diaphyseal axis, when following the axis of the femoral neck) (mm);
- Femoral offset (the distance between the centre of the femoral head and the closest point of the femoral shaft axis) (mm);
- Neck-shaft/collodiaphyseal angle (the angle between the axis of the femoral neck and the femoral shaft in the plane of the neck) (°);
- Mechanical tibiofemoral angle (the angle between the mechanical axis of the femur and the mechanical axis of the tibia in the frontal plane of the knee) (°);
- Sagittal tibiofemoral angle (the angle between the femoral and tibial mechanical axis in the sagittal plane) (°);
- Femoral mechanical axis-femoral shaft angle (the angle between the mechanical and anatomical axis of the femur in the frontal plane of the knee) (°);
- Femur mechanical angle (the angle between the femoral mechanical axis and the tangent of the femoral condyles medially) (°);
- Tibial mechanical angle (the angle between tibial mechanical axis and the tangent of the tibia plateau medially) (°);
- Femoral torsion (the angle between the femoral neck axis and the posterior condylar line projected on a plane perpendicular to the mechanical axis of the femur) (°);

- Tibial torsion (the angle between the transmalleolar and transcondylar axis projected on a plane perpendicular to the mechanical axis of the tibia) (°);
- Femorotibial rotation (the angle between the posterior condylar line of the femur and tibia);
- Sagittal pelvic tilt (the angle between the vertical axis and the axis linking the centre of the bicoxofemoral axis to the centre of the sacral plate) (°);
- Pelvic incidence (the angle between the perpendicular line to the centre of the sacral plate and the axis joining the centre of the sacral plate to the centre of the bicoxofemoral axis) (°);
- Sacral slope (the angle between the horizontal axis and the sacral plateau) (°);
- Lateral pelvic tilt (the distance between the upper edges of the acetabuli in the frontal plane view) (mm);
- Pelvis axial rotation (the angle between the bicoxofemoral axis and the detector's plane in the horizontal plane) (°).

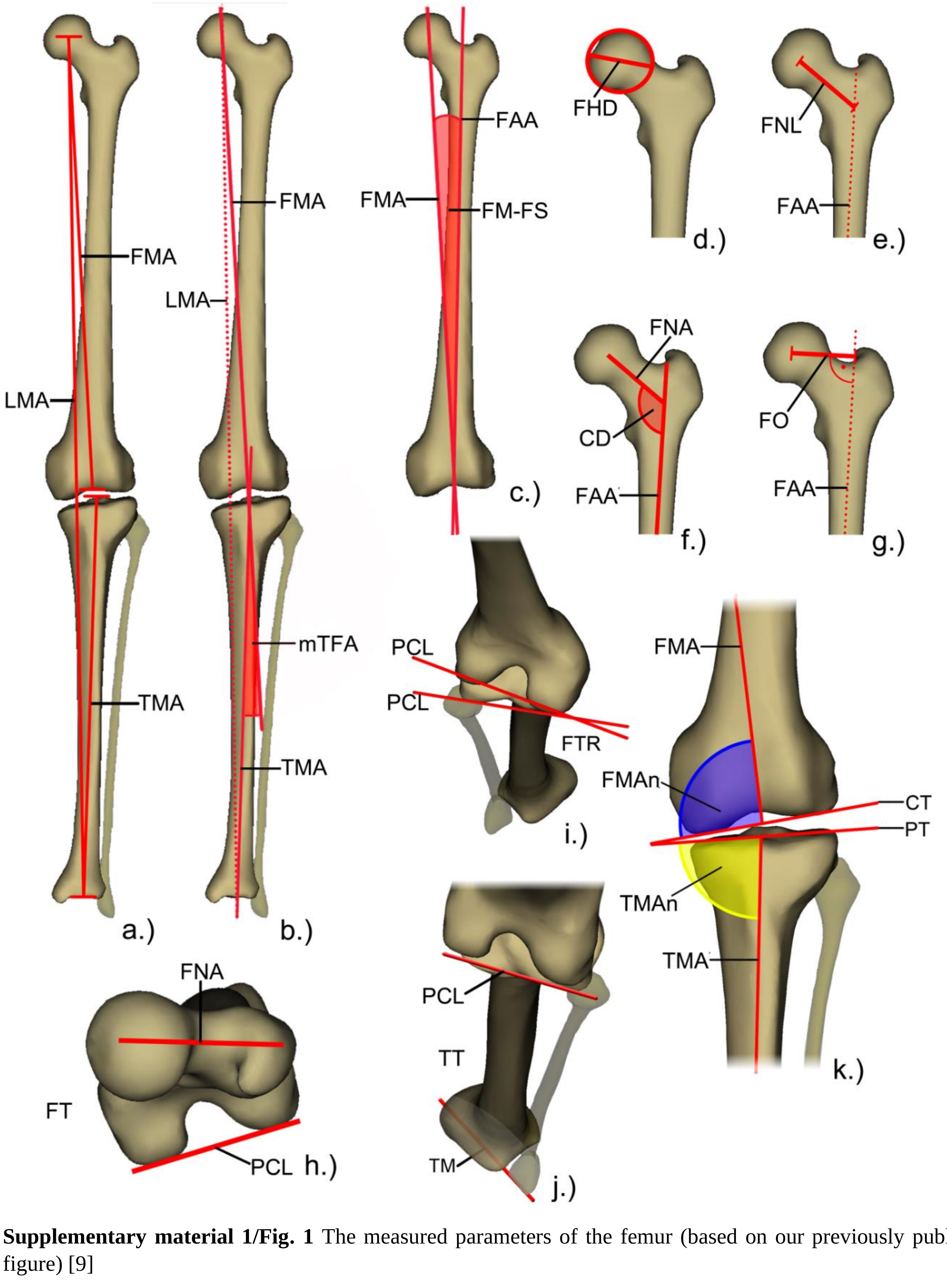

Supplementary material 1/Fig. 1 The measured parameters of the femur (based on our previously published figure) [9]

CD – Collodiaphyseal angle; FAA – Femur anatomical axis; FHD – Femoral head diameter; FMA – Femur mechanical axis; FMAn – Femoral mechanical angle; FM-FS – Femoral mechanical axis-femoral shaft angle; FNA – Femoral neck axis; FNL – Femur neck length; FO – Femoral offset; FT – Femoral torsion; FTR – Femorotibial rotation; LMA – Lower limb mechanical axis; mTFA – Mechanical tibiofemoral angle; PCL – Posterior condylar line; TM – Transmalleolar line; TMA – Tibial mechanical axis; TMAn – Tibial mechanical angle; TT – Tibial torsion.
