# Supplementary material for: Neck-shaft angle measurement in children: accuracy of the conventional radiography-based (2D) methods compared to 3D reconstructions
Source: Sci Rep. 2022 Oct 3;12:16494. doi: 10.1038/s41598-022-20832-1 (PMC9529964; doi:10.1038/s41598-022-20832-1)
Supplement: Supplementary file 3 — Supplementary Information 3. [file 41598_2022_20832_MOESM3_ESM.pdf]

Supplementary material 3.

	Biggest diameter - 1/3 femur	Circle fitting - 1/3 femur	Circle fitting - 1/2 femur	Circle fitting - full femur
Intraobserver reliability	0.972/0.977/0.975	0.962/0.966/0.962	0.959/0.961/0.980	0.968/0.978/0.969
Interobserver reliability	0.912	0.930	0.902	0.911

Supplementary material 3. Intra- and interobserver reliability of the 2D measuring methods (Cronbach's Alpha)
