# Supplementary material for: Neck-shaft angle measurement in children: accuracy of the conventional radiography-based (2D) methods compared to 3D reconstructions
Source: Sci Rep. 2022 Oct 3;12:16494. doi: 10.1038/s41598-022-20832-1 (PMC9529964; doi:10.1038/s41598-022-20832-1)
Supplement: Supplementary file 4 — Supplementary Information 4. [file 41598_2022_20832_MOESM4_ESM.pdf]

Supplementary material 4.

Age (year) n=24/year	Biggest diameter - 1/3 femur	Circle fitting - 1/3 femur	Circle fitting - 1/2 femur	Circle fitting - full femur	Femoral torsion
4	7.68 ± 3.25	6.05 ± 2.85	7.80 ± 4.74	7.71 ± 4.97	24.23 ± 9.76
5	7.75 ± 5.99	6.56 ± 4.50	8.05 ± 6.27	8.16 ± 5.86	24.57 ± 12.34
6	7.91 ± 5.61	8.02 ± 5.43	8.34 ± 5.57	8.63 ± 5.24	24.85 ± 13.49
7	5.43 ± 2.67	5.99 ± 3.42	6.37 ± 2.64	6.74 ± 3.10	24.56 ± 9.25
8	5.15 ± 4.50	5.53 ± 3.90	4.69 ± 3.15	5.30 ± 3.04	22.02 ± 9.59
9	4.68 ± 3.09	4.43 ± 3.00	3.48 ± 2.77	3.29 ± 2.82	22.71 ± 6.67
10	4.82 ± 3.98	5.38 ± 4.21	4.55 ± 3.23	5.05 ± 3.75	21.73 ± 10.08
11	5.98 ± 4.03	6.01 ± 4.05	4.90 ± 3.42	4.91 ± 3.02	20.51 ± 12.75
12	4.31 ± 3.41	4.75 ± 3.37	3.57 ± 2.50	3.79 ± 2.14	21.28 ± 10.23
13	4.87 ± 3.27	4.43 ± 3.11	3.68 ± 2.52	4.52 ± 2.21	21.63 ± 6.19
14	4.43 ± 3.30	4.35 ± 3.20	4.32 ± 2.52	4.20 ± 2.46	18.18 ± 11.48
15	5.58 ± 3.93	4.31 ± 3.14	3.02 ± 2.23	3.60 ± 1.90	19.80 ± 10.20
16	3.98 ± 3.27	3.71 ± 2.36	3.60 ± 2.75	3.98 ± 2.36	16.60 ± 11.72
**SUM (n=312)**	**5.58 ± 4.12**	**5.35 ± 3.80**	**5.11 ± 3.99**	**5.37 ± 3.87**	**21.78 ± 10.56**

Supplementary material 4 – Table 1. Average absolute difference between the 3D reconstruction and 2D measurement's results (mean ± S.D., degrees)

	True NSA (3D measurement)	Biggest diameter - 1/3 femur	Circle fitting - 1/3 femur	Circle fitting - 1/2 femur	Circle fitting - full femur	Femoral torsion
Male	129.79 ± 4.98	5.33 ± 4.13	5.36 ± 3.67	4.90 ± 3.66	5.21 ± 3.65	22.05 ± 10.69
Female	129.96 ± 5.21	5.83 ± 4.11	5.41 ± 3.94	5.30 ± 4.30	5.54 ± 4.09	21.51 ± 10.46
p	0.761	0.294	0.908	0.370	0.225	0.324

Supplementary material 4 – Table 2. Gender based differences in true NSA, absolute difference between the 3D reconstruction and 2D measurement's results and femoral torsion (average ± S.D., degrees), p value of independent sample T-test
