# Supplementary material for: Neck-shaft angle measurement in children: accuracy of the conventional radiography-based (2D) methods compared to 3D reconstructions
Source: Sci Rep. 2022 Oct 3;12:16494. doi: 10.1038/s41598-022-20832-1 (PMC9529964; doi:10.1038/s41598-022-20832-1)
Supplement: Supplementary file 5 — Supplementary Information 5. [file 41598_2022_20832_MOESM5_ESM.pdf]

Supplementary material 5.

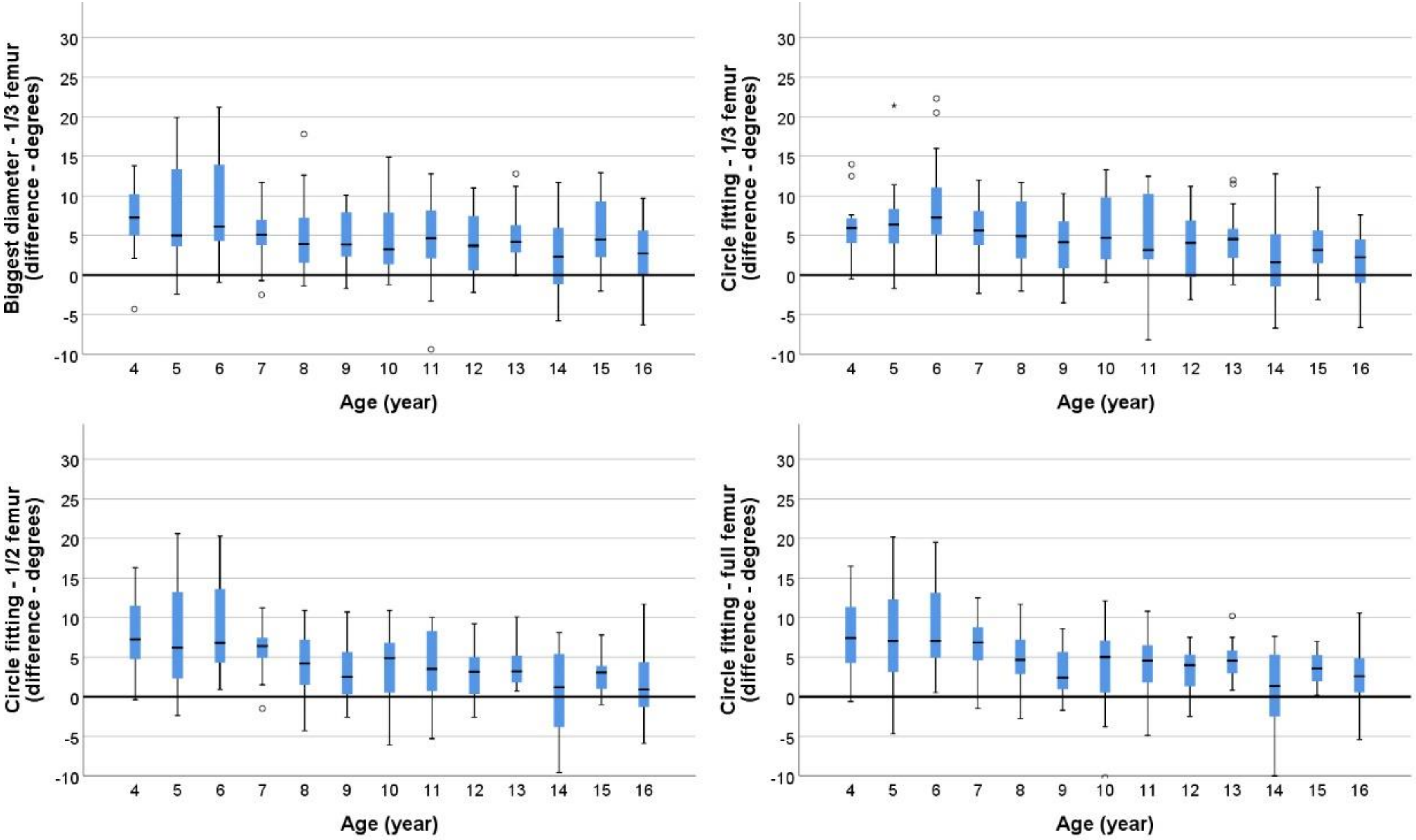

Supplementary material 5. Boxplot diagram of the difference between the 3D reconstructions and the 2D measurement results (°).
